# High-ICE and High-Capacity Retention Silicon-Based Anode for Lithium-Ion Battery

**DOI:** 10.3390/nano12091387

**Published:** 2022-04-19

**Authors:** Yonhua Tzeng, Cheng-Ying Jhan, Yi-Chen Wu, Guan-Yu Chen, Kuo-Ming Chiu, Stephen Yang-En Guu

**Affiliations:** Institute of Microelectronics, Department of Electrical Engineering, National Cheng Kung University, Tainan City 70101, Taiwan; m10506126@gmail.com (C.-Y.J.); el861223@gmail.com (Y.-C.W.); fb374208@gmail.com (G.-Y.C.); fibvpcbu3737@gmail.com (K.-M.C.); jespgl@gmail.com (S.Y.-E.G.)

**Keywords:** silicon, pyrolysis, LIB, anode, Super P, Ketjen black, initial coulombic efficiency, retention

## Abstract

Silicon-based anodes are promising to replace graphite-based anodes for high-capacity lithium-ion batteries (LIB). However, the charge–discharge cycling suffers from internal stresses created by large volume changes of silicon, which form silicon-lithium compounds, and excessive consumption of lithium by irreversible formation of lithium-containing compounds. Consumption of lithium by the initial conditioning of the anode, as indicated by low initial coulombic efficiency (ICE), and subsequently continuous formation of solid-electrolyte-phase (SEI) on the freshly exposed silicon surface, are among the main issues. A high-performance, silicon-based, high-capacity anode exhibiting 88.8% ICE and the retention of 2 mAh/cm^2^ areal capacity after 200 discharge–charge cycles at the rate of 1 A/g is reported. The anode is made on a copper foil using a mixture of 70%:10%:20% by weight ratio of silicon flakes of 100 × 800 × 800 nm in size, Super P conductivity enhancement additive, and an equal-weight mixture of CMC and SBR binders. Pyrolysis of fabricated anodes at 700 °C in argon environment for 1 h was applied to convert the binders into a porous graphitic carbon structure that encapsulates individual silicon flakes. The porous anode has a mechanically strong and electrically conductive graphitic carbon structure formed by the pyrolyzed binders, which protect individual silicon flakes from excessive reactions with the electrolyte and help keep small pieces of broken silicon flakes together within the carbon structure. The selection and amount of conductivity enhancement additives are shown to be critical to the achievement of both high-ICE and high-capacity retention after long cycling. The Super P conductivity enhancement additive exhibits a smaller effective surface area where SEI forms compared to KB, and thus leads to the best combination of both high-ICE and high-capacity retention. A silicon-based anode exhibiting high capacity, high ICE, and a long cycling life has been achieved by the facile and promising one-step fabrication process.

## 1. Introduction

Rechargeable lithium-ion batteries (LIB) are widely used in portable electronic devices, laptop computers, and electric cars. To meet the rising demand in storage capacity, silicon-based anode materials have been extensively studied. The advantages of abundant silicon with high theoretical capacity (3579 mAh g^−1^, Li_15_Si_4_) [1,2] and relatively low discharge potential (~0.4 V vs. Li/Li^+^) makes it the most promising anode material for next-generation LIB.

High-capacity silicon-based anodes suffer from inadequate cycling life and capacity retention due to excessive consumption of the limited supply of lithium in a packaged LIB. When a fresh anode reacts with the electrolyte to form solid-electrolyte-phase, which is necessary for subsequent stable and repetitive discharging and charging operations, part of the lithium is not recycled. The ratio of released lithium to the initially consumed lithium during the lithiation of silicon for the first time is the initial coulombic efficiency (ICE), that is typically around or less than 80% [3]. That means about 20% or more lithium is lost just by the discharging of the anode for the first time. When crystalline silicon forms silicon-lithium compounds, the volume increases by up to 300–400% depending on which compound it forms. When the volume changes, especially inhomogeneous volume changes in an anode, excessive stresses, cracks, and even pulverization of individual silicon particles occur. As a result, the fresh silicon surface is exposed to the electrolyte. The formation of SEI on the fresh silicon surface consumes additional lithium [4,5]. Therefore, in the following cycles, part of the lithium is lost, resulting in the coulombic efficiency being less than 100%. In other words, lithium consumption accumulates from cycle to cycle until there is inadequate lithium for normal operation of a LIB. Besides silicon, other contents of the anode, especially including the nanoscale conductivity enhancement additives, may cause irreversible catalytic decomposition of the electrolyte to form lithium-containing compounds. This adds to the permanent loss of lithium. 

Recently, we reported a promising and facile one-step process for the fabrication of a silicon-based anode, which exhibits high capacity and excellent capacity retention with a long cycling life. Pyrolysis of silicon flakes in CMC/SBR binders with Ketjen black (KB) conductivity enhancement additives converted the binders into a strong, porous, and graphitic carbon structure, which protected silicon from excessive reactions with the electrolyte and the possible loss of electrical contacts when silicon breaks into smaller and separated silicon pieces due to stresses generated by the volume changes of silicon. By the enclosure of a single piece of strong carbon structure made from the binders, broken silicon pieces are kept together by the surrounding graphitic carbon. Although the ICE was significantly improved by the pyrolysis process, the measured ICE value of 78% indicated that some contents in the anode consumed lithium during the first discharging half cycle [6]. In other words, some additives besides silicon flakes and binders are suspected to be of excessive reactivity with the electrolyte. Further work was carried out to investigate the effects of conductivity enhancement additives on the ICE and the capacity retention of the silicon-based anode after the pyrolysis. This paper reports the answer to the question of why the previous reported anode failed to demonstrate both high-ICE and high-capacity retention. 

Irreversible consumption of lithium can be reduced by the proper selection of conductivity enhancement additives, which are relatively inert to the electrolyte and the minimization of the initial effective surface area that needs to form SEI and subsequent creation of the fresh silicon surface due to cracking and pulverization of silicon particles. In this paper, effects of two kinds of conductivity enhancement additives including Super P and Ketjen black (KB) on the ICE will be studied. Compared to Super P, KB has a unique branched-chain structure and a very large specific surface area that is more than 20 times larger than that of Super P of the same weight [7,8]. An advantage of KB’s microstructure is its large number of electrical contact points of conductive carbon, forming more electrically conductive and uniform paths in the anode [9]. The unique nanoscale structure and large specific effective surface area might lead to enhanced reactivity with the electrolyte during cycling. 

On the other hand, the minimization of SEI that is needed for proper operation of the anode during the first and subsequent cycles is achieved by using silicon particles of a smaller exposed surface area and the prevention of cracks and pulverization of these silicon particles [10]. Larger silicon particles are better than smaller silicon particles because of the higher surface to volume ratio of smaller silicon particles. However, large silicon particles tend to break into smaller pieces more easily than smaller silicon particles. Therefore, there is an optimized and commonly used particle size for anodes of LIB in the order of 100 nm or smaller. In terms of the particle shape, flat particles are better than rounded particles and rounded particles without sharp points are better than those with them. Sharp points of a rounded particle attract higher lithium-ion current density during lithiation and form silicon-lithium compounds earlier than the remaining part of a silicon particle. Nonuniform formation of silicon-lithium compounds causes stresses inside silicon particles and possible breakage into multiple pieces. The breakage exposes a fresh silicon surface that needs to consume lithium to form SEI. A flat silicon particle exhibits a smaller effective surface area than multiple silicon particles of the same volume or weight. For example, a silicon flake of 100 × 800 × 800 nm has the same volume and weight as 64 silicon cubes of 100 × 100 × 100 nm but exposes 25% less silicon surface. Silicon flakes will be used for the anode study in this work. 

The volume changes when silicon forms silicon-lithium compounds are so significant that large silicon particles would crack or pulverize [11]. For smaller silicon particles, even when there are no cracks during the formation of silicon-lithium compounds for the first time, when the compounds decompose during the charging half cycle, a single crystalline silicon particle becomes amorphous. Stresses induced in the subsequent discharge–charge cycles might cause the amorphous silicon particles to break into multiple pieces. The breakage also causes SEI surrounding the silicon particle to crack, with the fresh silicon surface being exposed to the electrolyte. Porous anodes have been reported in the literature to preserve room for silicon volume expansion and to effectively reduce stresses and risks of particle pulverization. Silicon nanoparticles in core-shell structures with voids in between require expensive processes to manufacture, although they work well [12]. An economic manufacturing process is desirable.

Pre-treatment of silicon particles covers the silicon surface with layers of coatings which are inert to electrolytes, permeable to lithium, and conductive for electron transport. Silicon carbide, silicon nitride, silicon oxide, and graphitic carbons are among the coatings used on silicon particles, which have been reported to be effective in minimizing irreversible consumption of lithium and increasing ICE. These coating processes are time-consuming and in many cases are expensive, too [13,14,15,16].

Although proper coatings on silicon particles protect silicon from undesirable and irreversible reactions with the electrolyte, most coatings cannot sustain the stress due to volume changes of the silicon when it forms silicon-lithium compounds. Nonuniform coatings on individual silicon particles cause some silicon particles to expand in volume earlier than the others. The inhomogeneous volume expansion causes macroscopic cracks of the anode and the loss of electrical connections with the current collector. It is desirable to keep pulverized individual silicon particles together by an enclosure of adequate mechanical strength. One means of achieving this is to embed silicon particles in cavities of the bulk anode. Silicon will remain inside a cavity before and after pulverization. In short, the silicon particle, the protective coatings, the conductive coatings, and the binder are desired to be strongly bonded by conductive and chemically inert materials to the electrolyte in one porous solid anode. This will be the ultimate goal of this study. 

## 2. Materials and Methods 

### 2.1. Chemicals

AUO Crystal Corporation in Taichung City, Taiwan, supplied silicon flakes of about 100 nm-thick and 800 nm in length and width. It is a silicon wafer manufacturing company in Taiwan. The silicon flakes are part of silicon-containing waste slurry generated by cutting silicon ingots and from other silicon wafer manufacturing processes. The company uses an economic and proprietary chemical process to recover and purify the silicon flakes from the slurry. 

Battery-grade electrolyte, i.e., 1 M LiPF6 dissolved in equal volumes of ethylene carbonate (EC), diethyl carbonate (DEC), and dimethyl carbonate (DMC), with 10 wt.% fluoroethylene carbonate (FEC), was purchased from Taiwan Taipei Hopax Chems., MFG., Co. Ketjen black (KB) EC 600JD and Super P were obtained from Eubiq Technology Co. in Taipei, Taiwan, as conductivity enhancement additives.

### 2.2. Fabrication of Coin Half Cells with Si-Based Anode

Silicon flakes were mixed with conductivity enhancement additives (KB or Super P), sodium carboxymethyl cellulose (NaCMC), and styrene-butadiene (SBR) at a weight ratio of 70%:10%:10%:10%, respectively. The slurry was stirred homogenously, and then applied on a 10 μm-thick copper foil by means of a doctor blade. The thickness of the anode excluding the copper current collector was typically 15–20 µm. After the electrode was dried at 80 °C for 12 h, the electrode was cut into small pieces with a diameter of 12 mm. 

For pyrolysis of the anode, the anode was placed in a two-inch diameter quartz tubing. After an initial purge by argon (200 sccm), the reactor temperature was increased to 700 °C. It was held at that temperature for 60 min in argon atmosphere, and then the reactor was cooled down in argon at a rate of 5 °C/min.

For a comparison, anodes with only silicon flakes and binder (CMC/SBR), at a weight ratio of 70%:30% but without conductivity enhancement additives, were also fabricated. Both anodes of this composition with and without pyrolysis were fabricated. 

In order to verify the effects of the amount of KB additives to the anode performance, anodes with reduced KB additives of 5%, i.e., silicon flakes:KB:CMC:SBR = 75%:5%:10%:10%, instead of 10% in weight were also fabricated. When 5% KB was added, the silicon content increased from 70% to 75% in weight. 

The electrodes were placed into an Ar-filled glove box with residual oxygen and moisture contents of less than 0.5 ppm to assemble the coin cells. Lithium metal was used as the counter/reference electrode. The electrolyte was 1 M LiPF6 dissolved in equal volumes of ethylene carbonate (EC), diethyl carbonate (DEC), and dimethyl carbonate (DMC), and with 10 wt.% fluoroethylene carbonate (FEC).

### 2.3. Materials’ Characterization 

The morphology and structure of materials were observed by means of scanning electron microscopy (SEM, Hitachi-SU8000, Taipei, Taiwan) and scanning transmission electron microscopy (JEOL JEM-2100F Cs STEM, Taipei, Taiwan) with an acceleration voltage of 200 kV. A Horiba Scientific (Taipei, Taiwan) Raman system with a green laser at 532 nm and laser power at 450 mW was used to measure Raman spectra. The laser beam was focused on the sample surface in an area of about 10 µm in size. Raman spectra revealed the nanostructures of the sample. 

### 2.4. Electrode Characterization

The coin cells were disassembled in a glove box after 200 discharge–charge cycles. Thereafter, the cycled electrodes were immersed in anhydrous diethyl carbonate (DEC) to remove residual electrolytes and then dried in a vacuum drier at 60 °C for 8 h. Using transmission electron microscopy (JEOL JEM-2100F Cs STEM, Taipei, Taiwan) and scanning electron microscopy (Hitachi-SU8000, SEM, Taipei, Taiwan), the morphology and structure of the cycled electrodes were characterized.

### 2.5. Test Cells 

The charge–discharge cycling was analyzed by a battery testing system (BAT-750B, Taipei, Taiwan). The cells were cycled at a voltage window between 0.01 and 1.50 V vs. Li^+^/Li at 0.2 A/g for the first three cycles and 1.0 A/g for the following cycles. The specific capacity refers to the mAh per gram of silicon active material in the anode. 

Cyclic voltammetry (CV) and electrochemical impedance spectroscopy (EIS) were measured using Autolab (Metrohm AUTOLAB BV, Taipei, Taiwan). The CV measurement used a scanning rate of 0.1 mV/s at room temperature. The electrochemical impedance spectroscopy measurements were recorded in the frequency range of 0.01–100 kHz.

## 3. Results and Discussion

**Figure 1:**
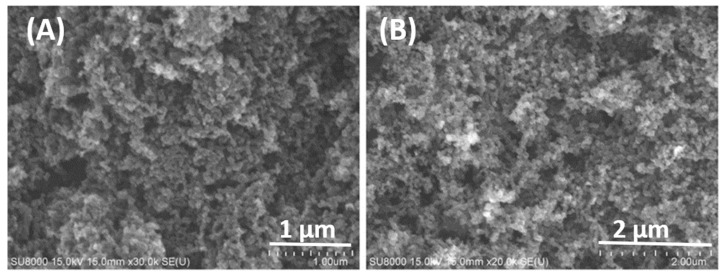
SEM image of (**A**) KB and (**B**) Super P conductivity enhancement additives.

**Figure 2:**
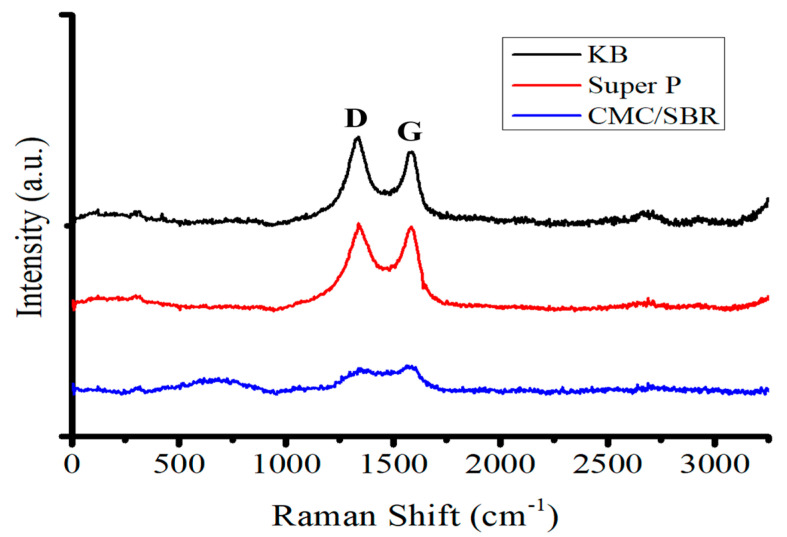
Raman spectra of carbon blacks of KB, Super P, and pyrolytic CMC/SBB.

Figure 1 shows SEM images of KB and Super P conductivity enhancement additives. The sizes of these nanoparticles are in the range of 50–100 nm. Figure 2 shows the Raman spectra of KB, Super P, and the pyrolyzed equal-weight mixture of binders CMC and SBR. The conductivity enhancement additives and the mixture of binders were analyzed by Raman spectroscopy before the fabrication of the anode. The Raman signal intensity ratio of the G-band to the D-band indicates the degree of graphitization. Super P exhibits a higher G/D ratio of 104% in comparison with that of 82.6% for KB. This is consistent with the XRD patent reported in [1], showing that Super P exhibits a higher and distinct graphite-like (002) characteristic peak than KB. The binder is the least graphitized. 

**After pyrolysis of the CMC/SBR binders, the I_D_/I_G_ ratio was approximately 0.94**. A wide, amorphous, carbon characteristic peak was situated between wavenumbers 500 and 1000 cm^−1^. An ordered/amorphous carbon porous carbon structure was formed post-pyrolysis.

**Figure 3:**
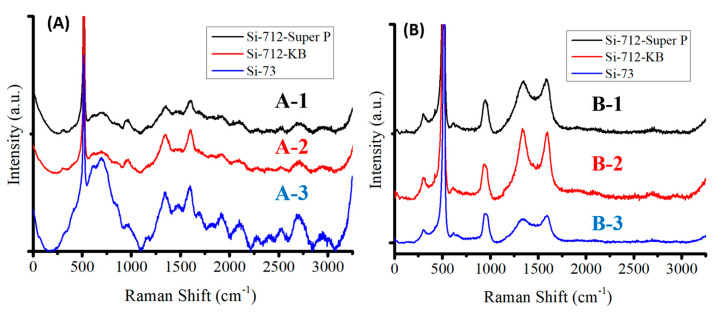
Raman spectra of (**A**) anodes made of (A-1) silicon:Super P:CMC/SBR = 70%:10%:20%, (A-2) silicon:KB:CMC/SBR = 70%:10%:20%, and (A-3) silicon:CMC/SBR = 70%:30%. (**B**) Anodes after pyrolysis and made of (B-1) silicon:Super P:CMC/SBR = 70%:10%:20%, (B-2) silicon:KB:CMC/SBR = 70%:10%:20%, and (B-3) silicon:CMC/SBR = 70%:30%. Raman excitation was performed by a 532 nm laser.

Silicon flakes were mixed with conductivity enhancement additives and the binder mixture for the fabrication of three sets of anodes. The compositions of these three sets of anodes were as follows: (1) silicon:Super P:CMC/SBR = 70%:10%:20%, (2) silicon:KB:CMC/SBR = 70%:10%:20%, and (3) silicon:CMC/SBR = 70%:30%. One specimen from each set of as-fabricated anodes was analyzed by Raman spectroscopy, with the Raman spectra shown in Figure 3A. The remaining anode was pyrolyzed in order to graphitize the binder for encapsulating silicon flakes and forming a porous carbon structure for the anode. The anode after pyrolysis was analyzed by Raman spectroscopy, with Raman spectra shown in Figure 3B.

Pre-pyrolysis electrodes consist of silicon flakes, conductivity enhancement additives, and binders. Carbon phases in the binders generate multiple peaks, as shown in the Raman spectra of Figure 3A, besides the G-peak, the D-peak, and the silicon-related peaks. After pyrolysis, various carbon phases in the binders were graphitized to different extents. The Raman spectra shown in Figure 3B exhibit mainly the G-peak, the D-peak, and silicon-related peaks. These anodes were used for capacity-voltage analyses. 

**Figure 4:**
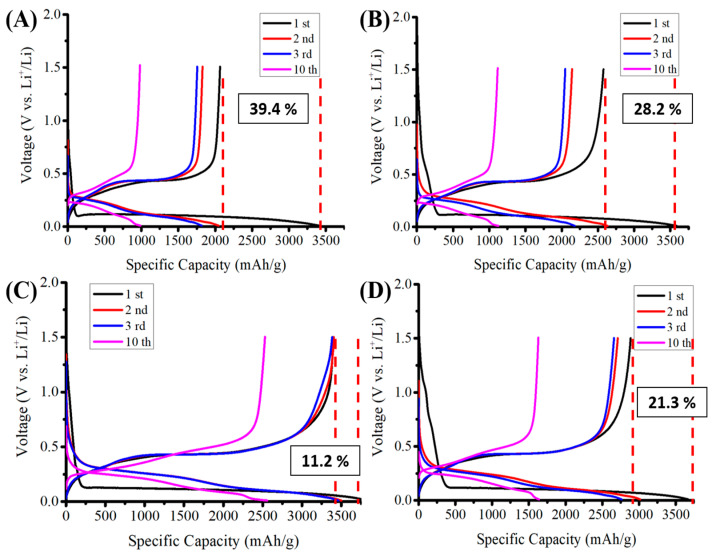
Capacity vs. voltage (Li^+^/Li) of anodes made of (**A**) silicon flakes:Super P:CMC/SBR = 70%:10%:20% by weight, (**B**) silicon flakes:KB:CMC/SBR = 70%:10%:20% by weight, (**C**) silicon flakes:Super P:CMC/SBR = 70%:10%:20% by weight after pyrolysis, and (**D**) silicon flakes:KB:CMC/SBR = 70%:10%:20% by weight after pyrolysis.

Figure 4 shows measured cyclic voltammetry (capacity-voltage) graphs of silicon-based anodes before and after pyrolysis with Super P or KB conductivity enhancement additives. These anodes correspond to anodes A-1, A-2, B-1, and B-2, which were used for Raman analyses, as shown in Figure 3. Figure 4A,B show the differences between Super P and KB before pyrolysis, while Figure 4C,D show the same after pyrolysis. The effects of pyrolysis on KB are shown by comparing Figure 4A with Figure 4C. Figure 4B,D shows the same for Super P.

When an anode discharges for the first time, the electrolyte reacts with active materials, where lithium will be stored until the anode is charged. A solid-electrolyte-phase (SEI) grows on the surface of the active materials. The discharge platform is attributed to monofluorinated carbon, which is mostly attributed to the decomposition product of fluoroethylene carbonate (FEC). A thin layer of SEI is first formed when the battery is discharged to 0.9 V (vs. Li/Li^+^) [17]. While the Si-based anode discharges further to 0.5 and 0.13 V, the SEI layer continues to grow and more SEI species are formed. After the formation is complete, the SEI layer consists of LiF, −CHF−OCO2-type compounds, carbonate/carboxylate, and ether-type species. 

During the subsequent charging half cycle, only part of the lithium is released to the electrolyte. Some of the consumed lithium is not restored. The ratio of the released lithium to the consumed lithium during the first cycle of discharging and charging the anode is known as the initial coulombic efficiency (ICE). A high ICE is important because the difference between 1, i.e., 100% coulombic efficiency, and the measured ICE is the percentage of the lost lithium used for the first discharging half cycle. For a packaged LIB, there is a limited amount of lithium, and when the accumulation of lost lithium reaches near the depletion of lithium, the discharge–charge functions of the LIB fail. Therefore, it is desired for the coulombic efficiency (CE) to be as close to 100% as possible. Due to the initial formation of SEI, the CE for the first cycle of the discharge–charge process is usually the lowest. Therefore, achieving a high initial CE (ICE) is an important indication for a LIB to achieve a long cycling life.

By comparing the cyclic voltammetry (capacity-voltage) graphs (Figure 4A,B) measured from anodes with Super P vs. KB conductivity enhancement additives, a much wider initial electrical discharge shoulder (side band) was clearly shown at 0.75~1.20 V (vs. Li/Li^+^) for anodes with KB than those with Super P. The KB additive induced more reactions between the anode and the electrolyte than the Super P additive did. By comparing the higher ICE for the anode with the Super P additive (88.8%) with that with KB (78.7%), the additional discharge reactions are believed to be irreversible lithium-containing compound formation reactions catalyzed by the KB additives between the electrolyte and the anode. 

By comparing the first cycle of the cyclic voltammetry (capacity-voltage) graphs in Figure 4A,C measured from anodes with Super P additives before and after pyrolysis, respectively, it showed that with Super P as an additive, the pyrolysis caused the ICE to increase significantly, from 100% − 39.4% = 60.6% to 100% − 11.2% = 88.8%. This is attributed to the pyrolysis process that creates graphitic carbon encapsulation of silicon flakes and the support of silicon flakes by porous graphitic carbon structure formed by the binders after pyrolysis. On the other hand, by comparing the first cycle of the graphs in Figure 4B,D measured from anodes with KB additives before and after pyrolysis, respectively, it showed that although the ICE was improved by the pyrolysis process, it only increased from 71.8% to 78.7%. Among the four anodes, the one with the Super P additive and after pyrolysis achieved the best ICE of 88.8%. It can be concluded that the Super P additive causes much less irreversible consumption of lithium from the electrolyte during the first cycle of the discharging process than KB additive does. This inertness of the Super P additive retains the high ICE produced by the pyrolysis process, which not only protects silicon flakes by graphitic carbon encapsulation but also provides a porous carbon structure for supporting the physical integrity of the silicon flakes and the anode. By comparing Figure 4C with the other graphs, it is clear that the pyrolyzed anode with the Super P conductivity enhancement additive achieved not only the highest ICE of 88.8% but also higher CEs of 97.3%, 97.6%, and 99.0% for the second, the third, and the tenth cycles of discharge and charge, respectively.

**Figure 5:**
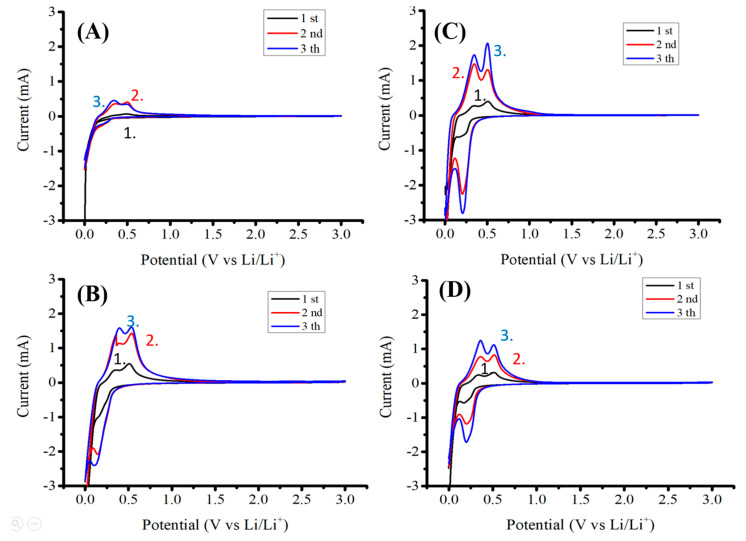
Cyclic voltammetry of anodes made of (**A**) silicon flakes:Super P:CMC/SBR = 70%:10%:20% by weight, (**B**) silicon flakes:KB:CMC/SBR = 70%:10%:20% by weight, (**C**) silicon flakes:Super P:CMC/SBR = 70%:10%:20% by weight after pyrolysis, and (**D**) silicon flakes:KB:CMC/SBR = 70%:10%:20% by weight after pyrolysis. The 1st, the 2nd, and the 3rd cycles are marked 1, 2, and 3 in the figures, respectively.

Figure 5 shows four discharge–charge cyclic voltammetry (CV) curves corresponding to the four capacity vs. voltage curves shown in Figure 4. The discharge begins with the lithiation of the crystalline silicon at near-zero volt. The lithiation forms lithium-silicon alloys. When alloys dissociate to release lithium, silicon is not recrystallized but instead becomes amorphous silicon. The de-lithiation curves exhibit two peaks near 0.4 and 0.6 V. These peaks are attributed to a two-step de-lithiation process, i.e., the anode first de-lithiates to become an intermediate lithium silicon alloy [8].

Most CV curves were similar except that for the anode without pyrolysis and made of 10 wt.% of Super P as a conductivity enhancement additive. The addition of 10 wt.% Super P was not enough to create sufficient conductivity for the anode, resulting in the relatively low currents in the curves shown in Figure 5A. After the pyrolysis of the anode, Figure 5C shows much higher currents for the same anode before pyrolysis, as shown in Figure 5A.

**Figure 6:**
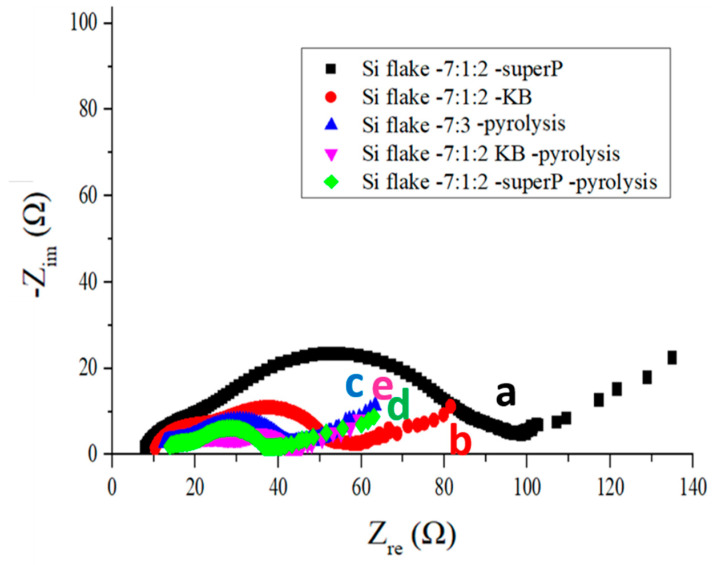
Electrochemical impedance spectra measured from anodes after the 200th cycle of discharge–charge: (**a**) (black square) anode made of silicon flakes:Super P:CMC/SBR = 70%:10%:20% in weight, (**b**) (red dots) anode made of silicon flakes:KB:CMC/SBR = 70%:10%:20% in weight, (**c**) (blue triangle) anode made of silicon flakes:CMC/SBR = 70%:30% in weight without a conductivity enhancement additive, (**d**) (pink inverted triangle) pyrolytic anode made of silicon flakes:KB:CMC/SBR = 70%:10%:20% in weight, and (**e**) (green diamond) pyrolytic anode made of silicon flakes:Super P:CMC/SBR = 70%:10%:20% in weight.

Figure 6 shows electrochemical impedance spectra (EIS) of as-fabricated anodes containing different conductivity enhancement additives before or after pyrolysis. An anode without the addition of a conductivity enhancement additive after pyrolysis is also presented for comparison. The anode without a conductivity enhancement additive was too resistive before pyrolysis and therefore is not presented with its EIS curve. All EIS curves were measured after the anodes completed the 200th cycle of the discharge–charge operation at a current rate of 1 A/g. The EIS measurement is effective in providing effective time-varying information about lithium-ion migration, carrier exchange, phase transfer, solid material transfer phenomenon, and the performance of the capacitance effect. It is an effective tool for observing the changes in the interface between the electrolyte and the electrode. The left side of each curve was measured at a higher frequency than the right side. The bottom left point on the horizontal axis represents the contact resistance. The radius of the EIS semicircle stands for the carrier transfer resistance, which reflects the rate of insertion of lithium-ions through the electric double-layer interface [18]. The slope of the oblique line in the low-frequency region on the right side of the curve is related to the diffusion rate of lithium-ions. The higher the slope of the oblique line is, the faster the diffusion rate of the sample [19].

Figure 6 compares the EIS curves measured from anodes after the 200th cycle of discharge–charge at the current rate of 1 A/g: (a) (marked in black square) anode with silicon flakes:Super P:CMC/SBR = 70%:10%:20% in weight, (b) (marked in red dots) anode with silicon flakes:KB:CMC/SBR = 70%:10%:20% in weight, (c) (marked in blue triangle) without a conductivity enhancement additive and with silicon flakes:CMC/SBR = 70%:30% in weight, (d) (marked in pink inverted triangle) pyrolytic anode with silicon flakes:KB:CMC/SBR = 70%:10%:20% in weight, and (e) (marked in green diamond) pyrolytic anode with silicon flakes:Super P:CMC/SBR = 70%:10%:20% in weight. From these curves, the effects of the pyrolysis process and the selection of conductivity enhancement additives are revealed.

The KB additive exhibits a very large specific surface area, which is effective in enhancing uniform electronic conductivity of a silicon-based anode and improving the electrochemical properties of the anode when compared with an equal-weight additive of Super P. However, KB is more reactive than Super P with the electrolyte, as shown by the voltage vs. capacity curves shown in Figure 4. A silicon-based anode without a conductivity enhancement additive is too resistive because of the high resistance of silicon. However, the pyrolysis of the as-fabricated anode without a conductivity enhancement additives converts the binder into graphitic carbon that not only enhances the conductivity of the anode but also provides protective encapsulation of individual silicon flakes and a porous carbon structure for assisting in the physical integrity of silicon flakes and the anode as a whole. The excellent electrochemical impedance curve that is comparable with anodes with both conductivity enhancement additives after the pyrolysis process is shown c in Figure 6, in comparison with d and e in Figure 6. The effects of conductivity enhancement additives on the cycling performance cannot be revealed by the EIS spectra alone. It will be evaluated in the subsequent capacity retention measurements.

As-fabricated silicon-based anodes before pyrolysis exhibit a lower contact resistance after 200 discharge–charge cycles than those after pyrolysis. However, the impedance in the low-frequency region is significantly larger than that of the pyrolytic anodes. This indicates that transportation of lithium-ions is hindered by the solid-electrolyte-phase in silicon-based anodes without pyrolysis. In addition, the charge transfer resistance of the anodes without pyrolysis is significantly higher than that of the pyrolytic electrodes [20]. It proved that the porous structure in pyrolytic anodes improved the permeability of the electrolyte. This is expected to be beneficial to the uniform lithiation and de-lithiation of an anode and helps preserve the physical integrity of the active materials and the whole anode.

**Figure 7:**
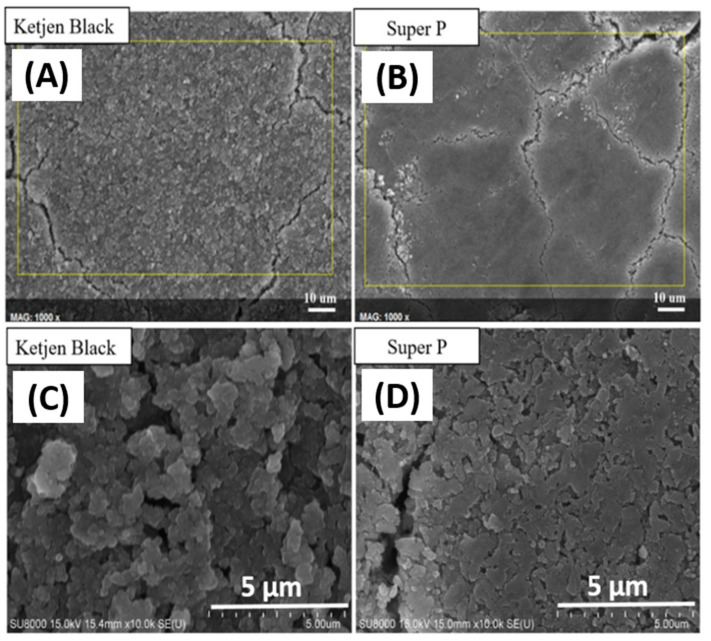
The SEM images of pyrolytic silicon-based anodes after the first discharge–charge cycle. Images (**C,D**) are enlarged images of (**A,B**), respectively. Images (**A,C**) are anodes with the KB additive. Images (**B,D**) are anodes with the Super P additive.

After the first discharge–charge cycle, the pyrolytic electrodes with a mass loading between 1.05 and 1.13 mg/cm^2^ were disassembled in a glove box, cleaned with DEC several times, and then dried. The morphology and elemental composition of the anodes were analyzed by SEM and EDS. Figure 7A,C show that the surface of pyrolytic anodes with the KB additive is much rougher than those with the Super P additive, as shown in Figure 7B,D after the first cycle. Cracks appeared on the anode and resulted in fast decay of the capacity when repetitive cycling was conductive. In contrast, the surface of the pyrolytic anode with Super P exhibited a smooth surface and maintained a better overall physical integrity.

**Figure 8:**
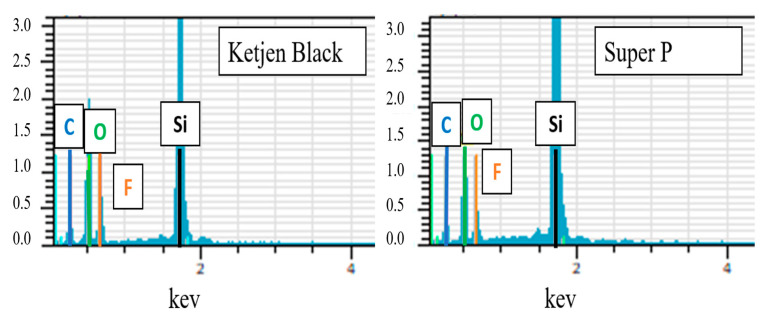
EDS spectra of the pyrolytic silicon-based anode with KB and that with Super P after the first discharge–charge cycle.

Figure 8 compares the EDS spectra of anodes with KB and Super P additives after the first discharge–charge cycle. As seen from the elemental analysis shown in Table 1, the contents of C, O, and F on the surface of the anode with the KB additive were much higher than those of the anode with Super P. These are the main elements of SEI compounds such as Li_2_O, LiF, Li_2_CO_3_, and Li_2_SiF_6_ [21]. The porous structure of KB with a high effective surface area promotes the catalytic formation of SEI, which consists of the multiple discharge platforms in the potential range of 0.2–0.8 V during the first discharge cycle, as shown in Figure 4. The much higher initial high capacity in this range is attributed to both reversible and irreversible reactions to form SEI layers. The large number of irreversible side reactions reduced the initial coulombic efficiency of the silicon-based anode with the KB additive. Besides, the thick SEI would aggravate the volume expansion of the silicon flakes, causing silicon flakes to lose contact with the conductivity agent and result in the decline in capacity.

**Figure 9:**
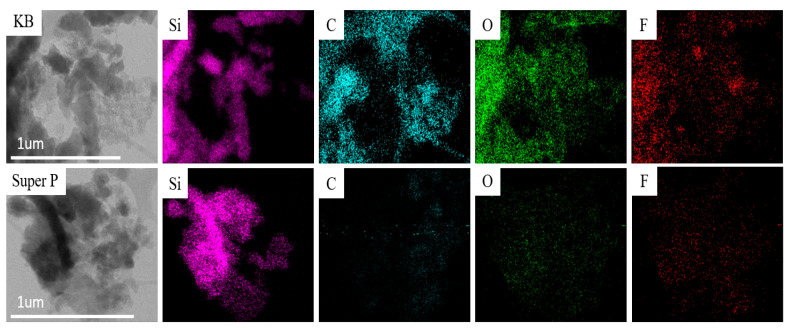
TEM images and EDS mappings of the Si-based anode with KB and that with Super P as conductivity enhancement additives.

Figure 9 shows TEM images and EDS mappings of the Si-based anode with KB and that with Super P as conductivity enhancement additives. The silicon mapping shows the portion of the TEM images where the silicon flakes are present. The anode with the KB conductivity enhancement additive apparently exhibited much stronger EDS signals from C, O, and F than that with Super P. This is attributed to reaction products between silicon and the electrolyte under the influence of the nanoparticles of conductivity enhancement additives. These reactions are not desirable because they consume additional lithium and cause the ICE to decrease. By comparing the fluorine elemental mapping for the anode with Super P and that with the KB additive, the anode with the Super P additive exhibited uniform F distribution while that with the KB additive exhibited nonuniform distribution. For the anode with the KB additive, there appeared to be considerable aggregation of fluorine. Fluorine was mainly on the surface of the interface, corresponding to the SEI. In contrast, for the electrode with KB, the fluorine distribution was nonuniform, and there was considerable aggregation of fluorine. The aggregation of fluorine may indicate reactions between Si and LiPF_6_ during the charge/discharge process, or additional formation of SEI that is induced by KB. There were stronger carbon, oxygen, and fluorine signals on the anode with the KB additive than that with Super P. This is consistent with the lower ICE that was measured for the anode with KB than that with Super P.

**Figure 10:**
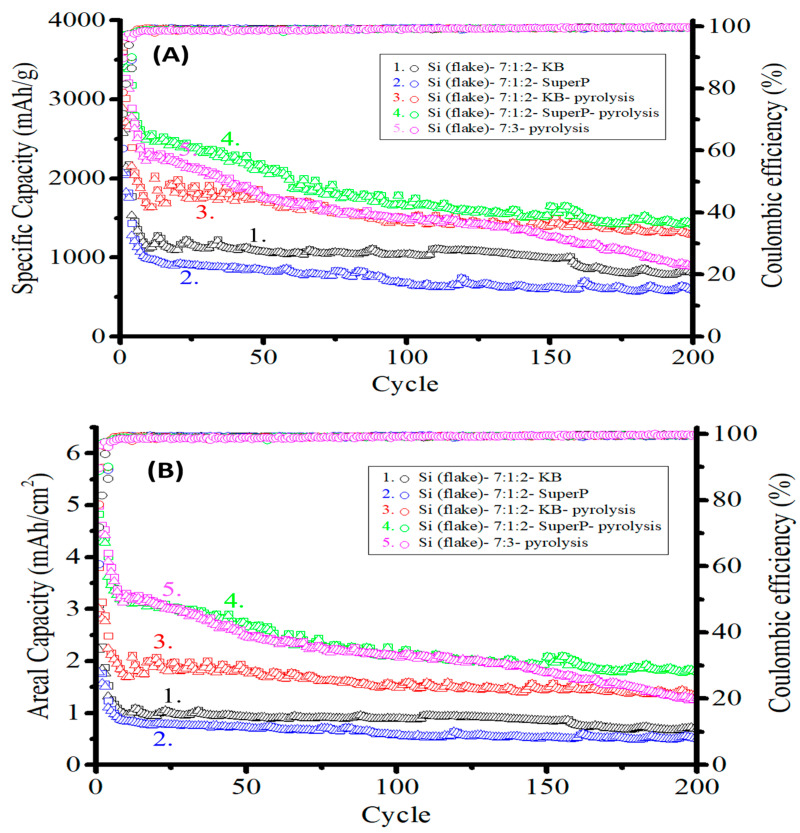
(**A**) Specific capacity and (**B**) areal capacity cycling performance of anodes made of: (1) (black) silicon flakes:KB:CMC/SBR = 70%:10%:20% in weight ratio without pyrolysis, (2) (blue) silicon flakes:Super P:CMC/SBR = 70%:10%:20% in weight ratio without pyrolysis, (3) (red) silicon flakes:KB:CMC/SBR = 70%:10%:20% in weight ratio after pyrolysis, (4) (green) silicon flakes:Super P:CMC/SBR = 70%:10%:20% in weight ratio after pyrolysis, and (5) (pink) silicon flakes:CMC/SBR = 70%:30% in weight ratio after pyrolysis but without a conductivity enhancement additive.

Figure 10A,B show the specific capacity and the areal capacity retention, respectively, of five sets of anodes made of: (1) (black) silicon flakes:KB:CMC/SBR = 70%:10%:20% in weight ratio without pyrolysis, (2) (blue) silicon flakes:Super P:CMC/SBR = 70%:10%:20% in weight ratio without pyrolysis, (3) (red) silicon flakes:KB:CMC/SBR = 70%:10%:20% in weight ratio after pyrolysis, (4) (green) silicon flakes:Super P:CMC/SBR = 70%:10%:20% in weight ratio after pyrolysis, and (5) (pink) silicon flakes:CMC/SBR = 70%:30% in weight ratio without a conductivity enhancement additive and after pyrolysis.

The mass loadings were in the range of 1.2–1.5 mg/cm^2^. The current of 0.2 A/g was used for the first three cycles, and 1 A/g was used for the remaining cycles. The ICE of the five electrodes were 71.8%, 60.6%, 78.7%, 88.8%, and 89.7%, as also shown in Table 2, respectively [22,23,24,25]. The anode without a conductivity enhancement additive but after being subjected to pyrolysis exhibited the highest ICE of 89.7%. The addition of Super P after pyrolysis resulted in a slightly lower ICE of 88.8%, which shows that Super P had little degradation effect on the ICE. In the contrast, the KB additive after pyrolysis reduced the ICE significantly from 89.7% to 78.7%, indicating that the KB additive results in more significant irreversible reactions of the electrolyte with active materials and an increased permanent loss of lithium. The pyrolysis process clearly enhances the ICE for anodes with or without conductivity enhancement additives.

Pyrolysis not only improves the ICE, but it also provides a strong and porous carbon structure where silicon flakes are embedded and uniformly encapsulated. A uniform enclosure of the active silicon flakes by a mechanically strong carbon structure is important in obtaining homogeneous alloying and de-alloying during discharging and charging half cycles. Homogeneous volume changes of silicon flakes reduce internal stress, which an anode may suffer from during non-homogeneous volume changes [26]. Silicon flakes encapsulated by the strong carbon structure made by pyrolyzed binders are retained inside the carbon structure when they form lithium-silicon compounds, and then the compounds dissociate to release lithium. Since individual silicon flakes are confined by the carbon structure, cracked and broken silicon pieces remain in contact with each other. The elastic carbon structure allows the volume occupied by silicon flakes to increase when silicon forms silicon-lithium compounds and provides pressure to keep the physical integrity of silicon flakes. Even when a silicon flake is broken into small pieces, the pressure provided by the strong carbon structure forces them to retain electrical contacts to each other. The capacity of silicon is, therefore, retained after many discharge–charge cycles.

Although the pyrolytic anode without conductivity enhancement additives exhibited excellent ICE, the conductivity of the anode was insufficient to maintain the long cycling performance of the anode. By comparing the cycling curves (3) and (4) for anodes after pyrolysis, curve (3) for the anode without a conductivity enhancement additive exhibited initial specific capacity and areal capacity, which decayed faster after 100 discharge–charge cycles. For long cycling, conductivity enhancement additives are still needed. The Super P additive performed better than the KB additive.

For an anode without conductivity enhancement additives, the binder-transformed graphitic carbon amounted to 5.7 wt.% of the anode after the pyrolysis process. The lowest carbon content also resulted in the highest ICE of 89.6% among all test samples. However, the gradually increasing internal resistance following repetitive discharge–charge cycles caused the capacity to decrease. After 200 cycles, the remaining specific capacity was only 900 mAh/g. The decrease in capacity is attributed to the non-homogeneous discharge and charge of the active silicon due to the nonuniform distribution of discharging and charging currents in the anode. The addition of conductivity enhancement additives contributed to the electrical conductivity of the anode and an improved uniformity of discharging and charging of actives in the anode.

By adding 10 wt.% of either KB or Super P, the electrical conductivity of the anode increased, and the non-homogeneous volume changes of the anode decreased. Cycling results show that both kinds of conductivity enhancement additives are beneficial to the capacity retention after cycling tests. After 200 cycles, the specific capacity of the anode with KB additives was approximately 1300 mAh/g, and the specific capacity of the anode with Super P additives was approximately 1400 mAh/g. KB has a far larger specific surface area compared to that of Super P. KB consumed a great deal of lithium-ions during the initial discharge–charge cycle, causing the ICE to be lower than that with Super P and that without additives. The ICE of the pyrolyzed anode with KB additives (78.7%) was approximately 10% lower than that of the pyrolyzed anode with Super P additives (88.8%) and the pyrolyzed anode without additives (89.7%).

Super P as a conductivity enhancement additive has a more ordered crystal structure and a lower specific surface area than KB. The ICE of a pyrolyzed anode with Super P additives was 88.8%. After 200 charge–discharge cycles, a capacity of 2 mAh/cm^2^ was retained.

**Figure 11:**
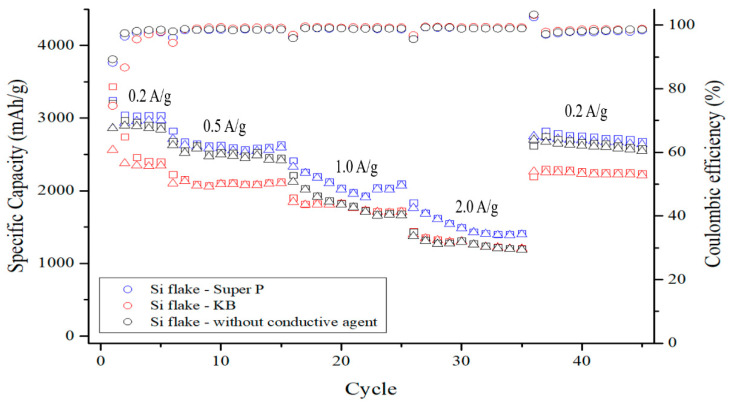
Cycling performance of pyrolyzed anodes made of: (1) silicon flake:Super P:binder = 70%:10%:20% by weight, (2) silicon flake:KB:binder = 70%:10%:20% by weight, and (3) silicon flake:binder = 70%:30% by weight at discharge–charge rates varied from 0.2 A/g (0.05 C) to 2 A/g (0.48 C). The discharge–charge rate is based on the weight of silicon flakes.

Figure 11 shows the discharge–charge rate effects on the cycling performance of pyrolyzed anodes without or with different conductivity enhancement additives. The mass loading of these three anodes was between 1 and 1.25 mg/cm^2^. Except for the anode with the KB additive, the initial coulombic efficiency was close to 90%. It can also be observed that the pyrolyzed anode with the Super P additive exhibited an excellent high capacity of 1420 mAh/g at the high discharge–charge current of 2 A/g. The addition of Super P with high crystallinity and a small specific surface area effectively reduced the rate of declining internal conductivity, while the porous carbon structures of the pyrolyzed binders joined forces to further improve the electrode polarization and minimize the initial irreversible capacity loss of the anode. The cycling life and capacity retention of pyrolyzed Si-based anodes were greatly enhanced.

**Figure 12:**
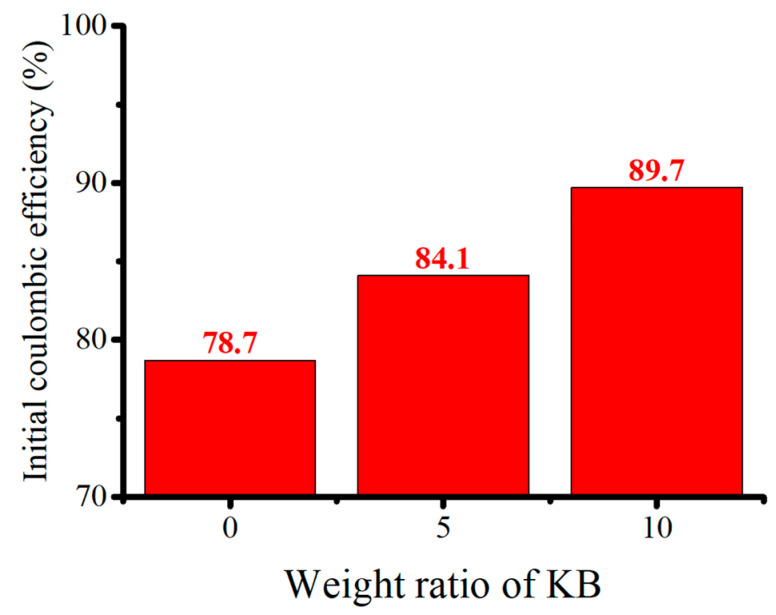
The relation between initial coulombic efficiency and mass ratio of Ketjen Black in the pyrolytic silicon-based electrode.

**Figure 13:**
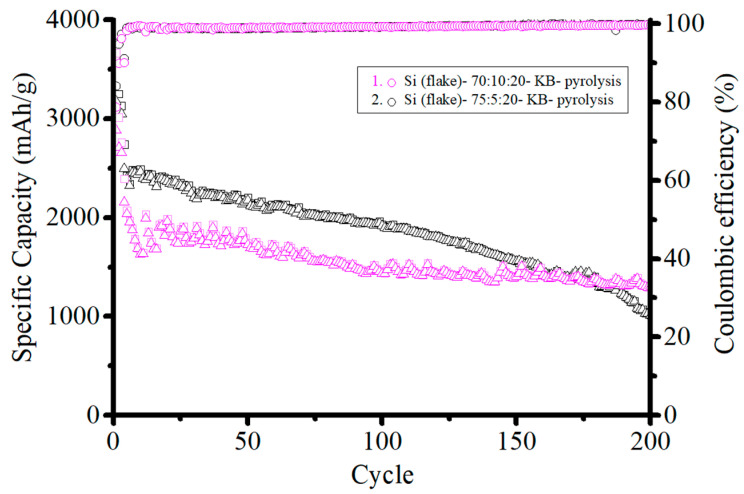
Specific capacity cycling performance of anodes made of: (1) (black) silicon flakes:KB:CMC/SBR = 75%:5%:20% in weight ratio, and (2) (pink) silicon flakes:KB:CMC/SBR = 70%:10%:20% in weight ratio after pyrolysis.

Although the ICE of the electrode with KB is poor, the capacity of the anode is stable at the discharge–charge currents of 1 and 2 A/g. This is attributed to the high electrical conductivity of KB. On the other hand, the pyrolyzed anode without conductivity enhancement additives had similar performance to the anode with Super P. However, without Super P, the anode cannot withhold the capacity due to increased internal resistance after a long cycling examination. When KB is to be used as the conductivity enhancement additive, the amount of KB needs to be reduced. Figure 12 shows the ICE of pyrolyzed anodes with 10%, 5%, and 0% KB additive by weight. The more KB is added, the lower the ICE will be. The ICE was 78.7%, 84.1%, and 89.7%, respectively. However, when a reduced KB of 5% by weight was added, the capacity retention was much worse than that with 10% by weight, as shown in Figure 13. The specific capacity of the pyrolyzed anode with 10 wt.% KB was 1450 mAh g^−1^ after 200 cycles. In comparison, only 1020 mAh g^−1^ was retained for the pyrolyzed anode with 5 wt.% KB after 200 cycles. This indicates that 5 wt.% KB is insufficient to cope with the increasing internal resistance during cycling.

**Figure 14:**
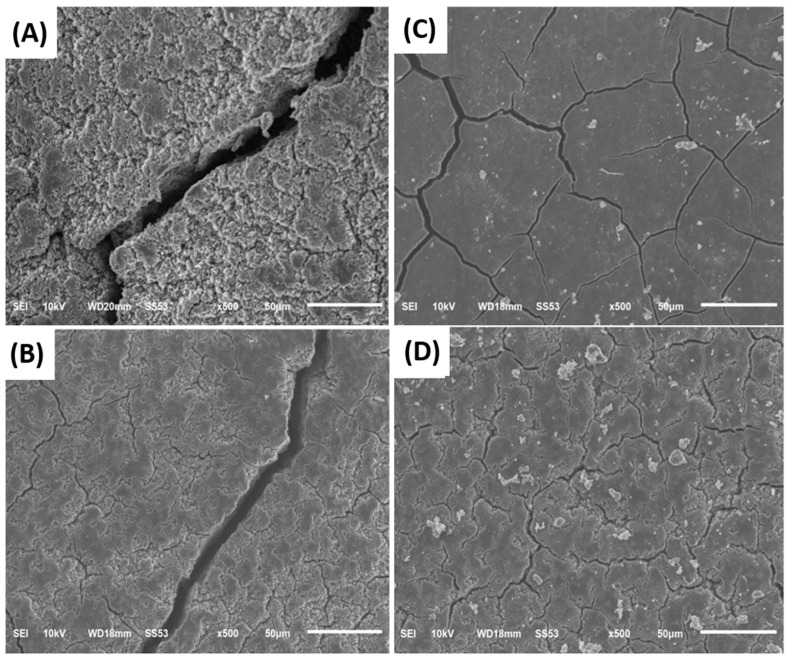
SEM image of the surface of cycled anodes made of: (**A**) Si:KB:binder = 70%:10%:20% by weight without pyrolysis, (**B**) Si:Super P:binder = 70%:10%:20% by weight without pyrolysis, (**C**) Pyrolyzed Si:KB:binder = 70%:10%:20% by weight, and (**D**) pyrolyzed Si:Super P:binder = 70%:10%:20% by weight. The anodes were cycled 200 times.

Figure 14A,B show the SEM images of the surface of silicon-based anodes with a weight ratio of Si:KB:CMC/SBR = 70%:10%:20%, and Si:Super P:CMC/SBR = 70%:10%:20%, respectively. The pyrolyzed silicon-based anodes are shown in Figure 14C,D. These anodes have been cycled in the discharge and charge process 200 times. The surfaces of cycled anodes without the pyrolysis treatment were severely cracked due to the volume changes. The surface of the anode with KB had more cracks than that with Super P. This is attributed to the fact that KB produces a thicker electrochemical passivation layer than Super P, especially during the first cycle. Furthermore, the network made with KB improved the overall conductivity of the anode and increased the usage of active materials, which aggravated and formed the severe surface damages.

On the other hand, the carbonized binder is not only beneficial to the overall electrical conductivity but also provides a conductive carbon film on the surface of the silicon flakes to reduce irreversible compound formation. Pores formed by the pyrolyzed binder provide extra space for silicon to expand and reduce the anode stress polarization, which in turn improves the physical integrity of the anode, as shown in Figure 14B,D. 

**Figure 15:**
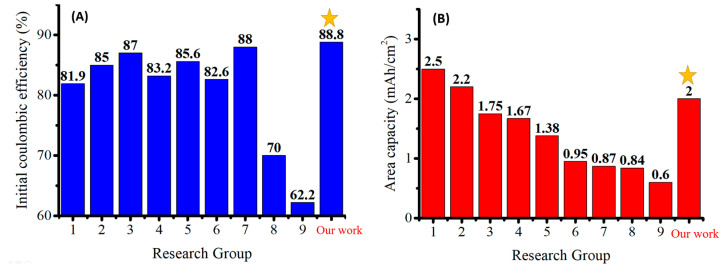
Comparison of (**A**) initial coulombic efficiency and (**B**) areal capacity of selected high-performance anodes reported in the literature. The one marked with star is this work.

We compared the pyrolyzed anode made in this work of Si:Super P:CMC/SBR = 70%:10%:20% by weight with recently published silicon-based anodes. Groups 1–9 correspond to [27,28,29,30,31,32,33,34,35]. Figure 15A compares the initial coulombic efficiency of anodes, and Figure 15B compares the areal capacity of anodes after 200 cycles of discharge and charge.

Group 1 volatilized copper at a high temperature to coat on nano-silicon powder. After that, a double-layer graphene was grown on the surface by chemical vapor deposition. The Si@BGra [27] was obtained after cleaning with nitric acid and water. Particles were loaded onto a Ni foam, which provides an effective 3D conductive network. After 200 cycles, the anode retained an area capacity of 2.5 mAh/cm^2^ [27].

Group 2 mixed the nano-silicon powder, graphite flakes, sucrose, and pitch together. The as-prepared powder was heated at 800 °C for 2 h in a quartz tube furnace under an Ar atmosphere to form the amorphous carbon coating layer on silicon powder. The resulting composite, C@SGG, was grounded for the fabrication of the anode with a weight ratio of active material:binder:Super P = 97%:2.5%:0.5% [28].

Group 7 coated SiO_2_ on the outer wall of carbon nanotubes by the sol-gel method. A layer of PVP was covered on the SiO_2_ layer afterwards. The prepared powder was mixed with metal aluminum and then placed in a furnace for sintering. During the sintering process, SiO_2_ was reduced to Si. The PVP on the outer wall transformed the CNT@Si@C [33] to a sphere with a diameter of about 5 μm. It increased the electrical conductivity and reduced the specific surface area at the same time [33].

Group 1 used nickel foam as the current collector. The thick porous structure increased the utilization of active materials per unit anode surface area and led to a very high areal capacity. Most of the active materials in the anode made by group 2 were carbon materials. The relatively stable lithium-ion migration mechanism improved the cycling performance of the high-areal-capacity anode. In addition, groups 1 and 2 used silicon nano-powder with sizes of less than 150 nm. This reduced the problem of silicon pulverization due to volume changes by cycling. Group 7 reduced the silica coating layer by using metal aluminum. The silicon layer as the active material can effectively improve the energy density. However, nanoscale silicon powder and complex manufacturing processes will greatly increase the costs of the battery cell.

## 4. Conclusions

Silicon flakes of about 100 nm-thick and 800 nm in length and width were recycled from silicon-containing waste slurry generated by fabricating silicon ingots and from other silicon wafer manufacturing processes. Silicon flakes were mixed with Super P, sodium carboxymethyl cellulose (NaCMC), and styrene-butadiene (SBR) in a weight ratio of 70%:10%:10%:10%. After the anode was pyrolyzed at 700 °C, the binder was converted to a graphitic carbon coating on the silicon flakes and an efficient 3D conductive network with a porous structure enclosing individual silicon flakes. The tight binding of the porous graphitic carbon structure with individual silicon flakes provided both protection of silicon against excessive irreversible reactions with the electrolyte leading to a high ICE and the desirable physical integrity of individual silicon flakes and the whole anode for high-capacity retention after long cycling. The significant effects of conductivity enhancement additives on ICE and long cycling performance were revealed. Super P was superior to KB when an equal weight was added to enhance the conductivity of the anode. Although KB provided better conductivity than Super P, it induced much more SEI formation during the first discharge–charge cycle than Super P did. As a result, a silicon-based anode with both a high initial coulombic efficiency near 90% and excellent capacity retention after a long cycling life has been demonstrated by means of a facile and one-step pyrolysis process, with Super P as the conductivity enhancement additive.

## Figures and Tables

**Table 1:** Relative elemental contents of the pyrolytic anode with KB and that with Super P conductivity enhancement additives.

Conductive Agent/Atomic Fraction (%)	C	O	F	Si
Ketjen Black	23.26	38.13	17.49	20.70
Super P	30.86	27.17	9.20	32.77

**Table 2:** Composition and respective electrochemical performance of silicon-based anodes.

Sample Numbers	Active Material	Pyrolysis	Binder	Weight Ratio (Active Material:Conductive Agent:Binder)	Mass Loading (mg/cm^2^)	Test Current (A/g)	ICE (%)
1	Si	No	CMC-SBR	70:10:20 (KB)	1.23	1	71.8
2	Si	No	CMC-SBR	70:10:20 (Super P)	1.23	1	60.6
3	Si	Yes	CMC-SBR	70:10:20 (KB)	1.23	1	78.7
4	Si	Yes	CMC-SBR	70:10:20 (Super P)	1.5	1	88.8
5	Si	Yes	CMC-SBR	70:30 (No conductive agent)	1.5	1	89.7
